# Glioblastoma recurrence correlates with NLGN3 levels

**DOI:** 10.1002/cam4.1538

**Published:** 2018-05-18

**Authors:** Rui Liu, Xing‐Ping Qin, Yang Zhuang, Ya Zhang, Hua‐Bao Liao, Jun‐Chun Tang, Meng‐Xian Pan, Fei‐Fei Zeng, Yang Lei, Rui‐Xue Lei, Shu Wang, An‐Chun Liu, Juan Chen, Zhi‐Feng Zhang, Dan Zhao, Song‐Lin Wu, Ren‐Zhong Liu, Ze‐Fen Wang, Qi Wan

**Affiliations:** ^1^ Department of Physiology Collaborative Innovation Center for Brain Science School of Basic Medical Sciences School of Medicine Wuhan University Wuhan China; ^2^ Department of Neurosurgery Renmin Hospital of Wuhan University Wuhan China; ^3^ Department of Radiology Renmin Hospital of Wuhan University Wuhan China; ^4^ Department of Neurology the Central Hospital of Wuhan Tongji Medical College of Huazhong University of Science & Technology Wuhan China; ^5^ Department of Physiology School of Basic Medical Sciences Hubei University of Medicine Shiyan Hubei China; ^6^ Department of Geriatrics Renmin Hospital of Wuhan University Wuhan China; ^7^ Institute of Neuroregeneration & Neurorehabilitation Department of Neurosurgery of the Affiliated Hospital Qingdao University Qingdao China

**Keywords:** ADAM10 inhibitor, deep brain region, glioblastoma, NLGN3, recurrence

## Abstract

Glioblastoma (GBM) is the most aggressive glioma in the brain. Recurrence of GBM is almost inevitable within a short term after tumor resection. In a retrospective study of 386 cases of GBM collected between 2013 and 2016, we found that recurrence of GBM mainly occurs in the deep brain regions, including the basal ganglia, thalamus, and corpus callosum. But the mechanism underlying this phenomenon is not clear. Previous studies suggest that neuroligin‐3 (NLGN3) is necessary for GBM growth. Our results show that the levels of NLGN3 in the cortex are higher than those in the deep regions in a normal human brain, and similar patterns are also found in a normal mouse brain. In contrast, NLGN3 levels in the deep brain regions of GBM patients are high. We also show that an increase in NLGN3 concentration promotes the growth of U251 cells and U87‐MG cells. Respective use of the cortex neuron culture medium (C‐NCM) and basal ganglia neuron culture medium (BG‐NCM) with DMEM to cultivate U251, U87‐MG and GBM cells isolated from patients, we found that these cells grew faster after treatment with C‐NCM and BG‐NCM in which the cells treated with C‐NCM grew faster than the ones treated with BG‐NCM group. Inhibition of NLGN3 release by ADAM10i prevents NCM‐induced cell growth. Together, this study suggests that increased levels of NLGN3 in the deep brain region under the GBM pathological circumstances may contribute to GBM recurrence in the basal ganglia, thalamus, and corpus callosum.

## 1 INTRODUCTION

Central nervous system (CNS) tumors are determined by their cellular origin and histopathological characteristics of the tissues.^1^, ^2^, ^3^ About 2% of all adult malignancies are brain tumors, of which 80% are gliomas.^4^, ^5^ Gliomas can occur at any age, regardless of ethnicity or gender and are classified into ependymoma, oligodendroglioma, astrocytoma, and oligoastrocytoma.^6^ World Health Organization (WHO) classification system classifies gliomas from grade 1 to grade 4. Grades 1 and 2 gliomas being referred to as low‐grade gliomas (LGG), while grades 3 and 4 gliomas are referred to as high‐grade gliomas (HGG).^7^, ^8^ Glioblastoma (GBM) belongs to HGG.

Glioblastoma is the most aggressive glioma of the brain, the average survival time is about 14 months after a surgery followed by radiotherapy and/or chemotherapy. A recurrence of GBM is almost inevitable in the short term.^9^ Surgery is usually the main treatment for GBM. The standard surgical method is to remove the largest amount of tumor while remaining a maximum neurological function.^10^ Preoperative assessment of GBMs relies on magnetic resonance imaging (MRI), which has the highest sensitivity to small tumors. It also provides an important reference for surgery and postoperative recurrence.^11^ In general, the lesions showed long T1 and long T2 signals on MRI, and were enhanced with mass or nodosity after contrast administration, most cases showed necrosis, hemorrhage, and cystic changes.

Neuroligins are synaptic cell‐adhesion molecules that interact with presynaptic neurexins to mediate transsynaptic signaling.^12^ Neuroligins have four major isoforms (neuroligin‐1 to 4),^13^ all of which act as ligands for presynaptic neuroxins, but perform different functions. Neuroligin‐1 is present in excitatory synapses, neuroligin‐2 in inhibitory synapses, Neuroligin‐3 (NLGN3) in both excitatory and inhibitory synapses, and neuroligin‐4 in glycinergic synapses.^14^, ^15^ Mostly, NLGN3 point mutation is associated with autism.^16^, ^17^, ^18^ Recent studies have shown that NLGN3 plays an important role in brain tumors, and the microenvironmental NLGN3 is necessary for glioma growth. By activating PI3K‐mTOR pathway, NLGN3 can promote the proliferation of gliomas. NLGN3 can also stimulate several oncogenic pathways, such as activation of focal adhesion kinase (FAK), and upregulation of several synapse‐related genes in gliomas cells.^19^, ^20^ The neuronligin‐3 is cleaved from both neurons and oligodendrocyte precursor cells (OPCs) via ADAM10 sheddase, and the ADAM10 inhibitors can prevent the release of NLGN3.^20^

In this study, we revealed the recurrence of GBM into the deep brain regions after tumor resection. In pathology situations, NLGN3 levels in the deep regions of the human brain are higher than those in the cortex, which is contrary to normal conditions. We further confirmed that high levels of NLGN3 promote GBM cell growth more rapidly in vitro. Together, high levels of NLGN3 in deep brain regions may play a key role in the induction of GBM recurrence after tumor resection.

## 2 MATERIALS AND METHODS

### 2.1 Human GBM and normal brain tissues

Brain specimens from male donators and patients are aged between 30 and 50 years. Human GBM tissues were obtained at the time of surgery from the Department of Neurosurgery in Renmin Hospital of Wuhan University, and tumor histology diagnosis was confirmed independently by three neuropathologists. The procurement of tissue usage and all image data for the study was obtained with written patient‐informed consent and approved by the Institutional Ethics Committee Faculty of Medicine at Renmin Hospital of Wuhan University (approval number: 2012LKSZ(010)H). The donators' brain materials had no clinical and post‐mortem neurological disease which were obtained from the Chinese Brain Bank Center (CBBC) by autopsy through a human body donation program, which is organized and implemented by the Wuhan Red Cross Society. According to the protocol of CBBC and the human body donation program, specific permission for brain autopsy and use of the brain material and medical records for research purposes were obtained either from the donators themselves or from their relatives, and was also approved by the Biomedical Research Ethics Committee of South‐Central University for Nationalities (approval number: 2017‐SCUEC‐MEC‐004).

### 2.2 Magnetic resonance imaging

The General Electric (GE) Company's Signa HDXt 3.0 T superconduct MRI scanner was used to take eight channel high‐resolution scans of the head‐neck coil, with axial baseline parallel corpus callosum body direction. The scanning sequences were performed on T1WI, T2WI and MRI enhancement.

### 2.3 Surgery

The surgical approaches were selected based on the lesion's characteristics, the safe entry zone, and both the ability and experience of the surgeons. Gross‐total resection was achieved in all patients, Electrophysiological monitoring of the cranial nerves, somatosensory, and motor function was used to help the surgeon.

### 2.4 Follow‐up and Clinical outcome

A retrospective study of 386 cases of GBM was conducted between 2013 and 2016, but for 320 cases were obtained in the follow‐up, which was conducted by an MRI scan at 1, 3, 6, 9, and 12 months after surgery.

### 2.5 Animals

Five adult male C57BL/6J mice were housed per cage, under a 12‐h light/dark cycle and a temperature‐controlled room (23‐25°C) with free access to water and food. The mice were allowed at least 7 days to acclimatize before experimentation. We used a total of 18 male mice in our in vivo experiments. Twelve adult pregnant female mice and 45 embryos were used in our experiments for the primary culture of cortical neurons. All animal use and experimental protocols were approved and carried out in compliance with the IACUC guidelines and the Animal Care and Ethics Committee of Wuhan University School of Medicine. Samples of the experimental groups were processed and assigned randomly.

### 2.6 Immunofluorescence analysis

Mice were treated with an over dose of isoflurane, then intracardiac perfusion with 0.9% saline, and 4% paraformaldehyde. The brains' tissues were then kept in 4% paraformaldehyde solution at 4°C overnight, transferred into 30% sucrose solution in 100 mol/mL phosphate buffer at 4°C for 72 hours. Brains' tissues were cut into 18‐μm coronal sections by a Leica VT1000S vibratome (Leica Micro‐systems AG, Nussloch, Germany). The human brain tissue slides were handled the same as well.

The immunofluorescence staining steps were based on the description of the prior execution.^21^ The brain sections were treated with primary mouse anti‐ NLGN3 (1:100) (sc‐137052) from Santa Cruz Biotechnology; the secondary antibody, goat anti‐mouse 488 from Molecular Probes (Eugene, USA); and the DAPI probe from Life Technologies. The tissue slides were photographed by Olympus fluorescent microscope (IX51, Olympus, Japan), analyzed using Image J Pro Plus 6.0 (Image J, USA).

### 2.7 Western blotting analysis

Western blotting was performed as previously described.^22^ Briefly, the polyvinylidene difluoride (PVDF) membrane by Millipore (USA) was used to incubate with the first antibody against AKT (Mouse, 1:1000) (#2920), phospho‐AKT (Ser473) (Rabbit, 1:1000) (#4060),Actin (Rabbit, 1:2000) (#8457) from Cell Signaling Technology (MA, USA), NLGN3 (Mouse, 1:500) (sc‐137052) that was from Santa Cruz Biotechnology. The recombinant protein neuroligin 3 was obtained from Novus biologicals (9069‐NL). The first antibodies were labeled with a secondary antibody, protein bands were imaged using SuperSignal West Femto Maximum Sensitivity Substrate (Pierce, Rockford, IL, USA). The EC3 Imaging System (UVP, LLC, Uplant, USA) was used to obtained blot images directly from the PVDF membrane. The data of Western blot were quantified using Image J Pro Plus 6.0 software.

### 2.8 Cell culture

U251 cells and U87‐MG cells were purchased from the Chinese Academy of Sciences Cell Bank. U251 cells, U87‐MG cells, and GBM001‐009 cells were harvested in a 3‐cm petri dish in DMEM supplemented with 10% heat‐inactivated FBS, penicillin G (100 U/mL), streptomycin (100 mg/mL), and L‐glutamine (2.0 mmol/L) and incubated at 37°C in a humidified atmosphere containing 5% CO_2_ and 95% air.

### 2.9 Cell count

The number of cells was counted using a counting plate. First, wipe the counting plate with anhydrous ethanol, then, use a silk cloth to wipe both the counting plate and cover glasses, and cover the top of the counting plate. Pipette 0.4% trypan blue dye solution into the cell suspension in a 1:1 ratio. Slowly drip from the edge of the counter, so that it fills the gap between the counter and cover. The count plate was observed by a low magnification (10 × 10 times). According to the calculation of the cells in the counting plate the square of 4 (each large square is divided into 16 small squares) within the cell number, counting only intact cells. In a large box, if there is a cell on the line, it is common to count the offline cells regardless of the online cells and to count the left‐side cells excluding the right‐line cells. The double counting error should not exceed ±5%. Microscopic observation is those that have strongly refraction and not colored are living cells, and those infected with blue are dead cells. After counting, the number of cells per milliliter of suspension is calculated. The number of cells can be calculated as follows: cell suspension cell number/mL = total number of 4 large cells/4 × 10 000.

### 2.10 Statistics

All data are expressed as mean ± SE. Newman‐Keuls tests were used for post hoc comparisons when appropriate. Student's *t* test and variance analysis were used to evaluate differences among groups. *P < *.05 was considered statistically significant.

## 3 RESULTS

### 3.1 Glioblastoma recurrence in the deep brain regions after tumor resection

A retrospective study of 386 cases of GBM was conducted between 2013 and 2016. These 320 cases have been followed up for more than 1 year. Moreover, 66 patients were not followed up, the reason for their withdrawal was either death, missing, or other reasons (Table 1). To our surprise, we found a meaning outcome that the primary regions of GBM are mainly located in the cortex (including frontal lobe, temporal lobe, parietal lobe, and occipital lobe). Recurrence often occurs in deep regions of the brain, including basal ganglia, thalamus, and corpus callosum.

**Table 1:** Statistics of GBM patients

Recurrence Primary (386)	Basal ganglia (71)	Thalamus (35)	Corpus callosum (58)	Basal ganglia + Thalamus (60)	Basal ganglia + Corpus callosum (79)	Basal ganglia + Thalamus + Corpus callosum (17)	Losing follow‐up (66)
Frontal lobe (116)	18	13	15	19	22	7	22
Temporal lobe (98)	26	14	15	10	12	4	17
Parietal lobe (87)	16	5	14	15	23	3	11
Occipital lobe (85)	11	3	14	16	22	3	16

**Figure 1:**
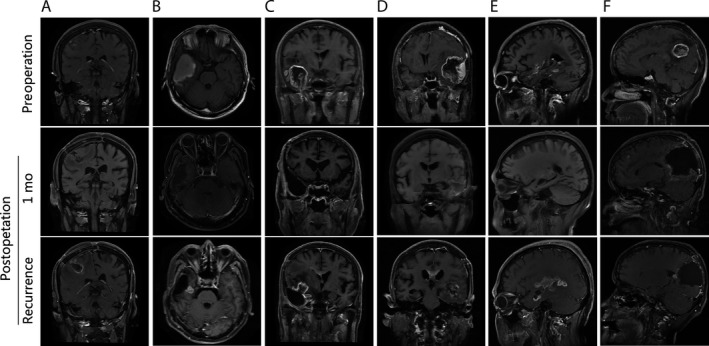
GBM recurrence in the deep brain regions after tumor resection. All the tumors are located in the cortex: A, Temporal lobe. B, Temporal lobe. C, Frontal lobe. D, Temporal lobe. E, Temporal lobe. F, Parietal lobe. After the operative, the tumors were completely removed at the 1st month of follow‐up, but the tumor in the fundus of the operative regions recurred during the 3rd month

In Figure 1A, the patient was accompanied by intractable epilepsy and the medication was ineffective. Therefore, we have performed a tumor resection surgery after the definite diagnosis. Fortunately, epilepsy was effectively controlled in the postoperative period and the tumor was also completely removed at the 1st month of follow‐up, but the tumor in the fundus of the operative regions recurred during the 3rd month. In Figure 1B, the patient had a right temporal lobe tumor, the solid mass density was not significant, and had cystic changes without a significant enhancement. During the follow‐up period, the tumor was completely removed one month after operation. There was no contrast enhancement in the operation region. However, when we performed an MRI examination 3 months after operation, we have found an abnormal enhancement signals in the deep surgical region, suggesting recurrence. Similar features were also found in Figure 1C‐F. In Figure 1C, the contrast enhancement was obvious and recurrence was multi‐originated, but the direction of extension was directed to the same place, named basal ganglia. In Figure 1D, the recurrence of tumor invaded the corpus callosum. Figure 1E,F shows recurrence invading thalamus.

### 3.2 Glioblastoma developing into deeper brain regions as time goes on after recurrence

We further studied the characteristics of this phenomenon. We found out that the trend of tumor recurrence into deep brain regions happens gradually over time.

**Figure 2:**
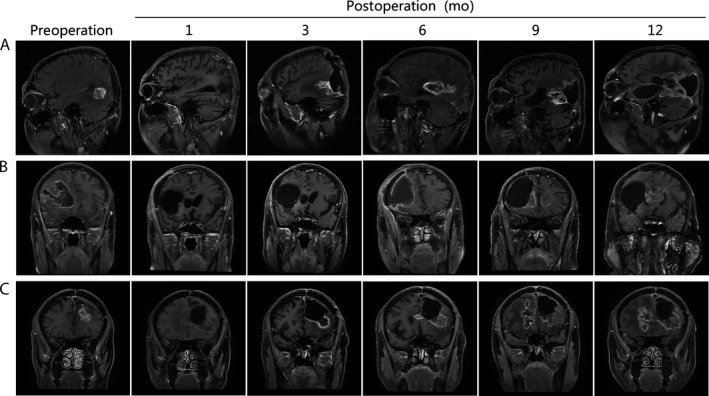
GBM developing into deeper brain regions as time goes on after recurrence. All the tumors are located in the cortex in pre‐operation: A, Frontal lobe. B, Temporal lobe. C, Occipital lobe. After the operative, the tumors were completely removed at the 1st month of follow‐up, but the tumor in the fundus of the operative regions recurred during the 3rd month. Moreover, the tumors invade in the deep brain region as time goes on: A 3, B 3, C 3, the tumors recurred in the fundus; A 6, corpus callosum; A 9, A 12, the other side of the brain; B 6, B 9, C 6, C 9 Basal ganglia; B 12, C 12 corpus callosum

In Figure 2A, the patient with GBM in the occipital lobe was completely resected in 1st and 3rd month's follow‐up, but in the 6th month, a ring enhancement was found, confirming tumor recurrence. Invasiveness of the tumor has first occurred within 9 months in the lateral ventricle wall, and in basal ganglia and corpus callosum within the 12th month. In Figure 2B, the patient was accompanied by occipital lobe GBM. At the 1‐month follow‐up, total resection was performed, but after 3 months, recurrence has occurred in the deep surgical regions and the temporal lobe was invaded. Over time, the invasiveness of tumor has expanded to the lateral wall of the ventricle during the 6th month, the thalamus during 9th month, and the basal ganglia and the corpus callosum during 12th month. In Figure 2C, the patient developed GBM in left frontal lobe. The tumor was completely removed within 1 month of surgery. However, after 3 months, the recurrence occurred in the deep surgical regions, which refers to the deep fundus of frontal lobe. Similarly, the tumor expanded to corpus callosum within 6 months, even worse, the tumor invaded the right frontal lobe within 9 months, and progressed in the lateral ventricle wall within 12 months.

### 3.3 Neuroligin‐3 in the deep brain regions is higher than in the cortex in the pathological conditions of GBM

**Figure 3:**
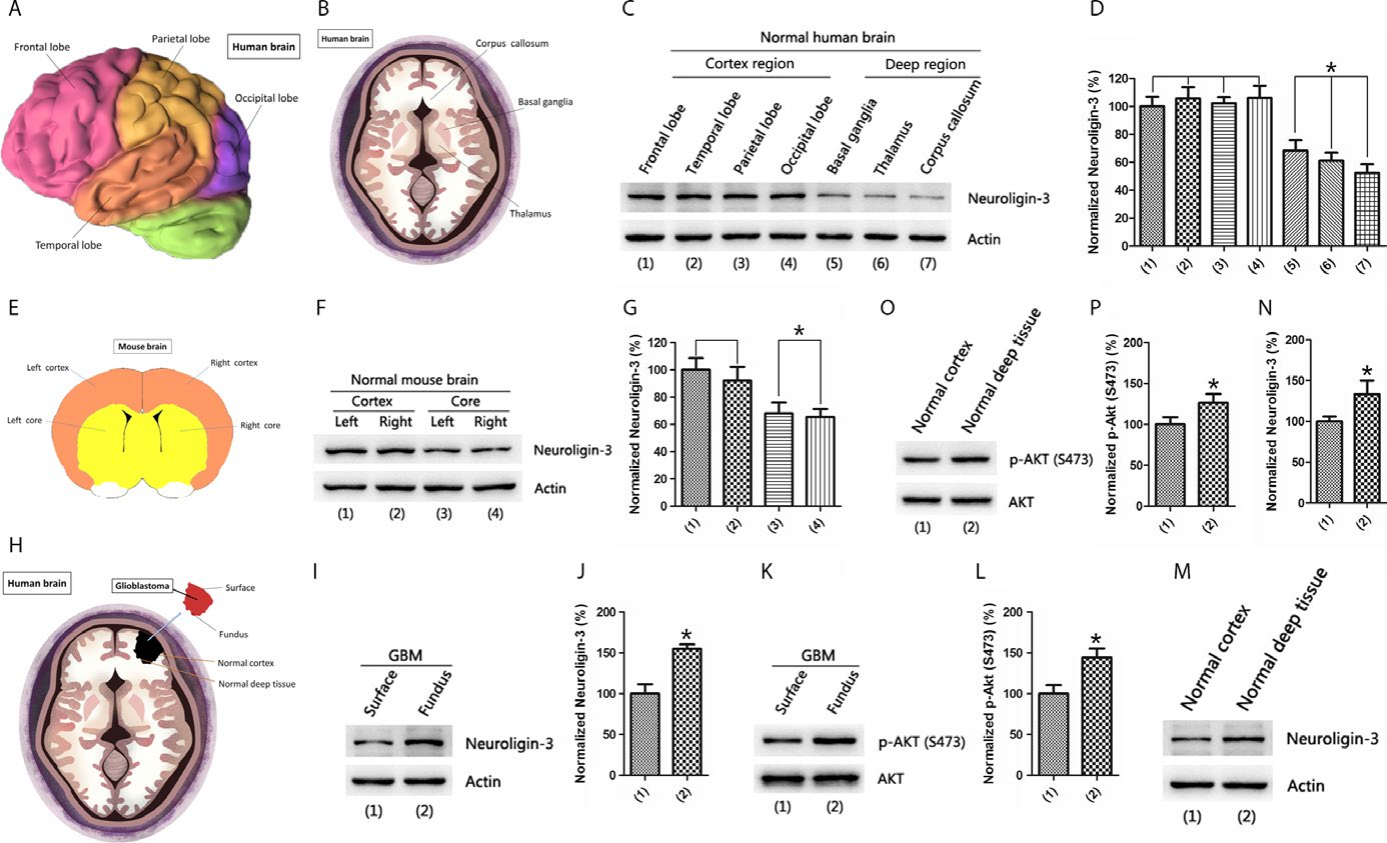
NLGN3 in the deep brain regions is higher than in the cortex in the pathological conditions of GBM. (A and B) Diagram shows the dissected structural domain of frontal lobe, temporal lobe, parietal lobe, occipital lobe, basal ganglia, thalamus, and corpus callosum in normal human brain. (C and D) Western blotting analysis of NLGN3 levels in the normal human brain frontal lobe, temporal lobe, parietal lobe, occipital lobe, basal ganglia, thalamus, and corpus callosum (C), quantification analysis of NLGN3 levels (D) shows a higher NLGN3 levels in the cortex (n = 5, *P < .05 vs the cortex). (E) Schematic diagram showing the dissected structural domain of cortex and core in normal mouse brain. (F and G) Western blotting analysis of NLGN3 levels in the normal mouse brain cortex and core (F), quantification analysis of NLGN3 levels (G) shows a higher NLGN3 levels in the cortex (n = 6, *P < .05 vs the cortex). (H) Schematic illustration showing the surface and fundus structural domain of GBM tissue from patients, the normal cortex and normal deep brain regions anatomical schematic in the GBM patients brain. (I‐L) Western blotting analysis of NLGN3 (I) and p‐AKT(S473) (K) levels in the surface and fundus of GBM tissues, quantification analysis of NLGN3 (J) and p‐AKT(S473) (L) levels shows a higher NLGN3 and p‐AKT(S473) levels in the fundus region (n = 3, *P < .05 vs the surface region). (M‐P) Western blots showing the NLGN3 (M) and p‐AKT(S473) (O) levels in the normal cortex and normal deep brain regions from GBM patients after surgery, quantification analysis of NLGN3 (N) and p‐AKT(S473) (P) levels shows a higher NLGN3 levels in the normal deep brain region (n = 3, *P < .05 vs the normal cortex). The data are expressed as mean ± SE. Statistical analysis was implemented by student's *t* test and variance analysis

In the Figure 1, we found that GBM recurred in the deep brain regions after tumor resection. Furthermore, in Figure 2 we noticed that GBM would grow to deeper brain regions over time after its recurrence. Previous studies have demonstrated that microenvironmental NLGN3 is essential for the growth of GBM.^20^ Therefore, we hypothesized that NLGN3 plays an important role in the recurrence of GBM. First, we have analyzed the levels of NLGN3 in the normal human brains (Figure 3A,B). Western blot results showed that the NLGN3 in the cortex was higher than in the deep brain regions (Figure 3C,D). Then, in the brain of normal mice (Figure 3E), we found a similar human NLGN3 expression pattern (Figure 3F,G). In the Table 1, the statistical results showed that most of the primary GBMs are mainly located in the cortex.

**Figure 4:**
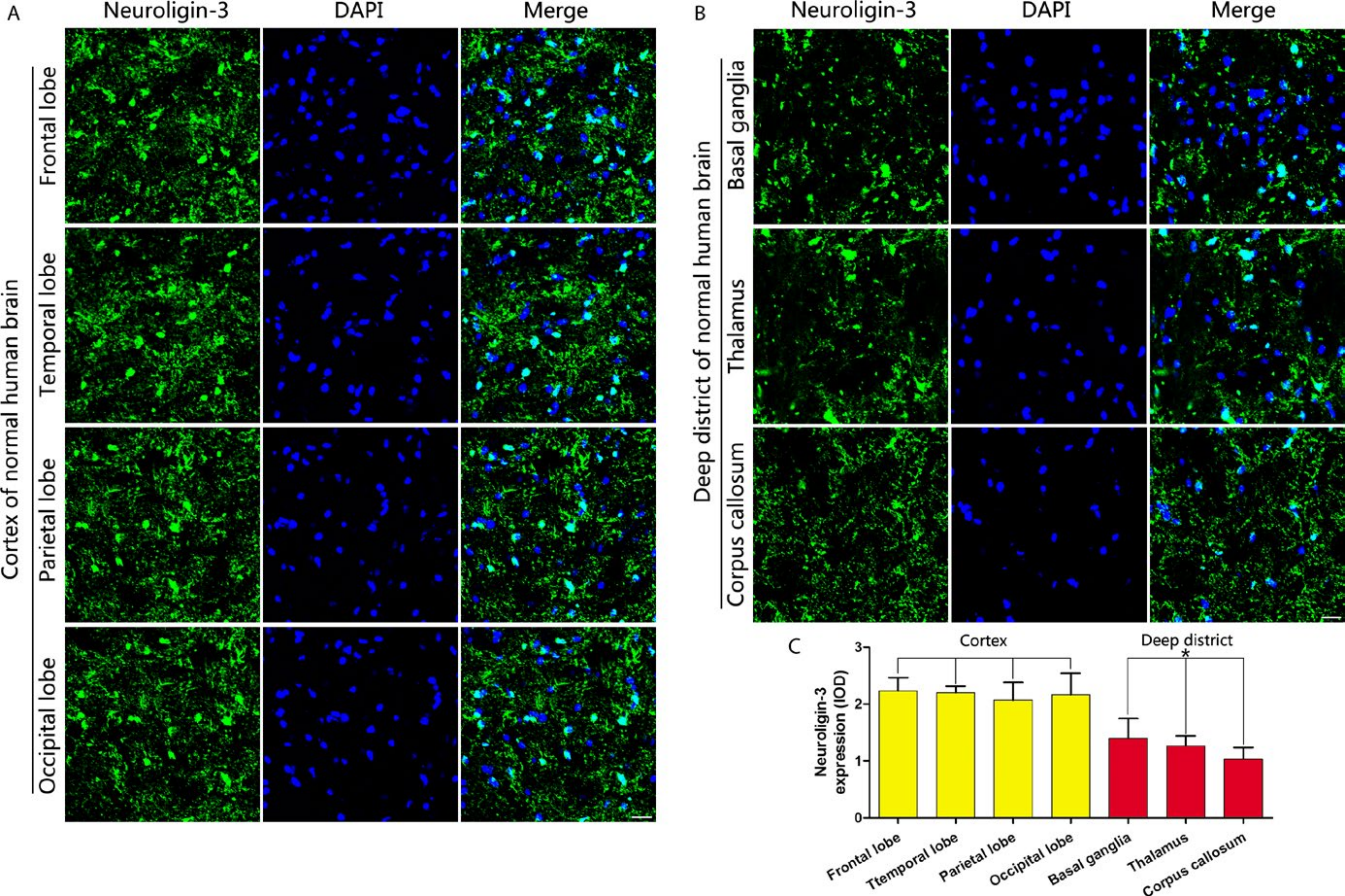
NLGN3 in the deep brain regions is lower than in the cortex in the normal human brain. (A and B) Immunofluorescence staining analysis of NLGN3 in the normal human brain frontal lobe, temporal lobe, parietal lobe, and occipital lobe (A); basal ganglia, thalamus, and corpus callosum (B). Scale bar, 20 μm. (C) The optical density (OD) analysis of NLGN3 expression (n = 5, *P < .05 vs the cortex). The data are expressed as mean ± SE. Statistical analysis was implemented by student's *t* test and variance analysis

**Figure 5:**
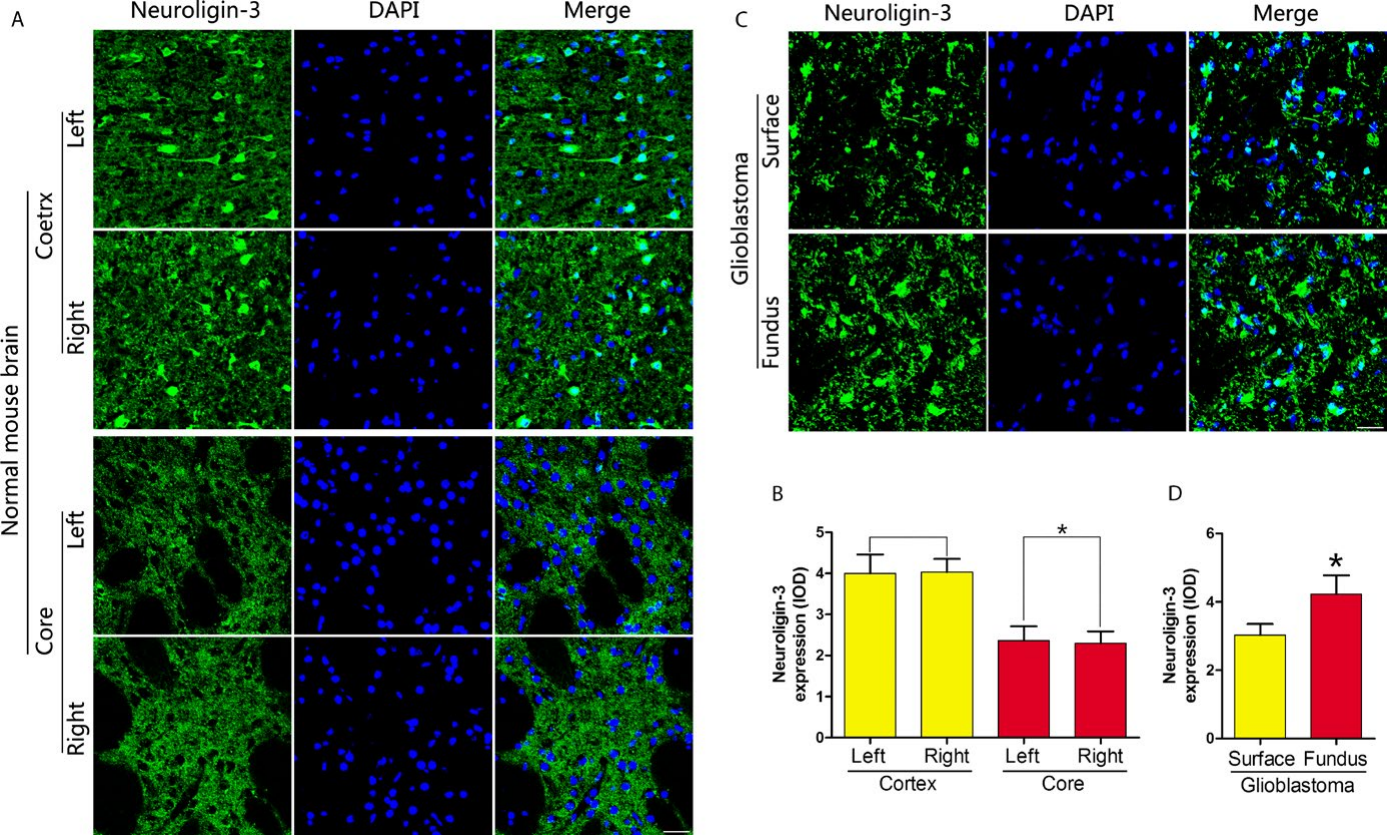
NLGN3 in the deep brain regions is higher than in the cortex under the GBM pathological circumstances. (A and B) Immunofluorescence staining analysis of NLGN3 in normal mouse brain cortex and core (A), the optical density (OD) analysis of NLGN3 expression (B) (n = 6, *P < .05 vs the cortex). Scale bar, 20 μm. (C and D) Immunofluorescence staining of NLGN3 in the two stage of GBM tissues (C), the optical density (OD) of NLGN3 expression (D) (n = 3, *P < .05 vs the surface region). Scale bar, 20 μm. The data are expressed as mean ± SE. Statistical analysis was implemented by student's *t* test and variance analysis

We further studied the level of NLGN3 under pathological conditions and analyzed the level of NLGN3 in GBM tissue from patients after tumor resection (Figure 3H). In the Figure 3I,J, we found that the level of NLGN3 was higher in the fundus region of GBM tissue compared to that in the surface region. Previous studies have shown that NLGN3 promotes the proliferation of gliomas by activating the PI3K‐mTOR pathway.^20^ Therefore, we further tested the level of p‐AKT (S473) in the GBM tissue and found that the level of p‐AKT (S473) was higher in the fundus region (Figure 3K,L). The standard procedure for surgery is to remove the largest tumor while remaining a maximum neurological function. The concept of tumor recurrence is the regrowth of the tumor at the primary site after tumor treatment subsided. In other words, recurrence means that tumor has appeared on the primary site, which considered normal after surgery. Furthermore, in Figure 3M,N, Western blot was used to analyze the levels of NLGN3 in normal deep brain regions and normal cortex under pathological conditions; results showed that NLGN3levels were higher in normal deep brain regions. We also have observed a similar pattern at the p‐AKT (S473) level (Figure 3O,P). Furthermore, using immunofluorescence staining analysis of NLGN3 in the normal human brain (Figure 4A,B), the optical density (OD) of NLGN3 expression showed in Figure 4C, normal mouse brain (Figure 5A,B), and GBM tissues from surgery (Figure 5C,D), we obtained a similar expression pattern to that of Western blot results.

Together, most of the primary GBMs are located in the cortex. Most of the GBMs recurrence are in the deep brain regions after surgery. Under normal conditions, the cortical NLGN3 levels are high. Under pathological conditions, a reversed expression pattern is performed. NLGN3 levels may be a potential determinant of GBM recurrence.

### 3.4 High levels of NLGN3 cause glioma cells to grow faster

**Figure 6:**
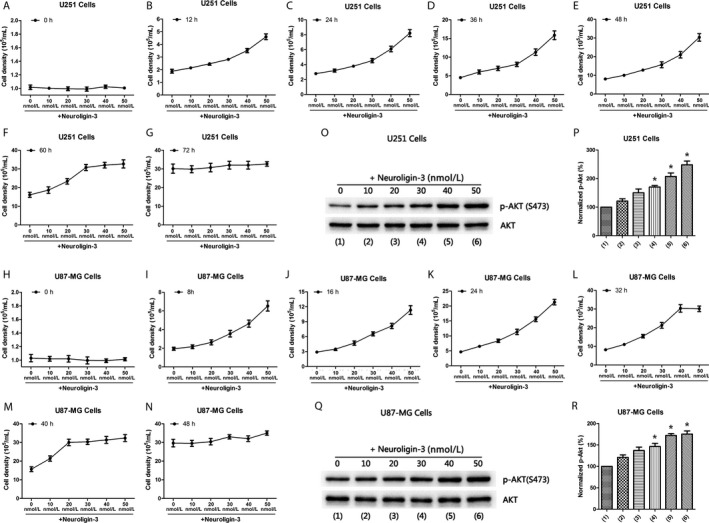
High levels of NLGN3 cause glioma cells to grow faster. (A‐G) Cell count analysis of U251 cells in 0 h (A), 12 h (B), 24 h (C), 36 h (D), 48 h (E), 60 h (F), and 72 h (G) after treatment with different concentration of NLGN3 (0, 10, 20, 30, 40, 50 nmol/L) (n = 6 independent cultures). (H‐N) Cell count analysis of U87‐MG cells in 0 h (H), 8 h (I), 16 h (J), 24 h (K), 32 h (L), 40 h (M), and 48 h (N) after treatment with different concentration of NLGN3 (0, 10, 20, 30, 40, 50 nM) (n = 6 independent cultures). (O and P) Western blots analysis of p‐AKT (S473) level (O) in U251 cells 48 h after treated with NLGN3 (0, 10, 20, 30, 40, 50 nmol/L); quantification analysis of p‐AKT level (P) shows an increased expression of normalized p‐AKT compared with 0 nM group (n = 6 independent cultures, *P < .05 vs the 0 nmol/L). (Q and R) Western blots analysis of p‐AKT (S473) level (Q) in U87‐MG cells 32 h after treated with NLGN3 (0, 10, 20, 30, 40, 50 nmol/L); quantification analysis of p‐AKT level (R) shows an increased expression of normalized p‐AKT compared with 0 nmol/L group (n = 6 independent cultures, *P < .05 vs the 0 nmol/L). The data are expressed as mean ± SE. Statistical analysis was implemented by student's *t* test and variance analysis

To further investigate whether higher levels of NLGN3 can affect the growth of GBM, we used NLGN3 recombinants to expose NLGN3 in vitro GBM cell lines, including U251 cell line and U87‐MG cell line. The U251 and U87‐MG cells were harvested in a 6‐cm diameter petri dish and the cell density was maintained at 1.0 * 10^5 ^± 0.2 * 10^5^/mL. Meanwhile, they were treated with different concentrations of NLGN3 recombinants (10, 20, 30, 40, and 50 nmol/L). Under normal conditions, it takes 3 days for U251 cells to grow from 1.0 * 10^5 ^± 0.2 * 10^5^/mL to a maximum density of 3.0 * 10^6 ^± 0.5 * 10^6^/mL, and U87‐MG cells takes 2 days. Therefore, we measured cell density of U251 cells at 12, 24, 36, 48, 60, and 72 hours after the harvesting of cells, and U87‐MG cells were at 8, 16, 24, 32, 40, and 48 hours. We found that the cell growth after NLGN3 exposure was faster than that of the control group in U251 cells (Figure 6A‐G). The group of NLGN3 (50 nmol/L) first reached its maximum density at 48 hours after harvesting (Figure 6E). U87‐MG cells showed similar characteristic pattern in the growth of U251 cells (Figure 6H‐N). U87‐MG cells firstly reached their maximum density at 32 hours after harvesting in the NLGN3 (50 nmol/L) group (Figure 6L). These results indicate that NLGN3 can promote the growth of glioma cells, and higher levels of NLGN3 can cause glioma cells to grow faster. Furthermore, Western blotting analysis of p‐AKT in U251 cells (Figure 6O) and in U87‐MG cells (Figure 6Q) at 48 and 32 hours was performed after harvesting of cells, respectively. Results showed that p‐AKT levels were upregulated after NLGN3 exposure and positively correlated with NLGN3 levels in U251 cells (Figure 6P) and U87‐MG cells (Figure 6R), which further supports our results in Figure 5A‐N.

### 3.5 Cortical neurons produce more NLGN3 than basal ganglion neurons to promote the growth of glioma cells

**Figure 7:**
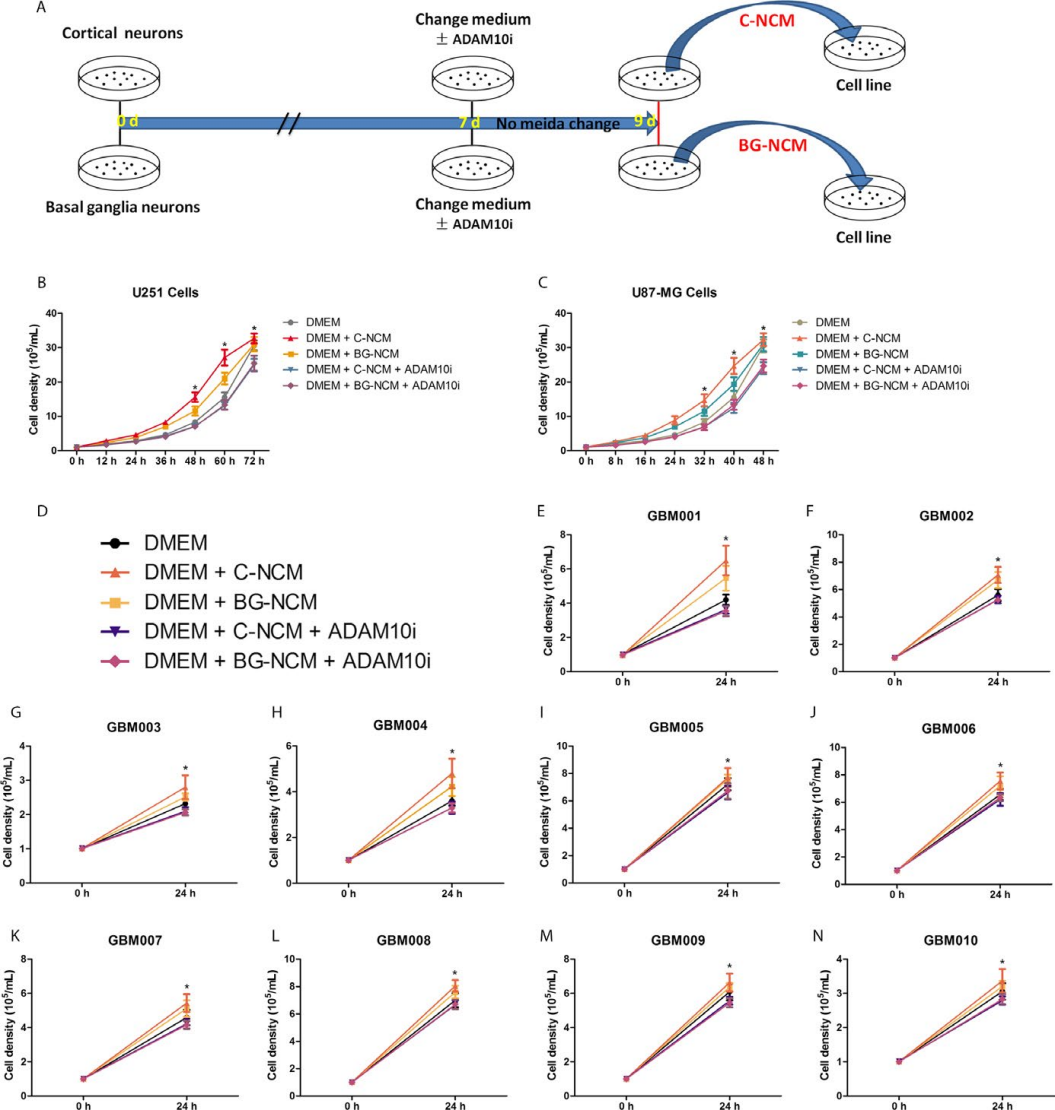
Cortical neurons produce more NLGN3 than basal ganglion neurons to promote the growth of glioma cells. (A) A time point's schematic diagram shows the procedure of cortex and basal ganglia primary culture neuron, the ADAM10i treatment handing time, and the C‐NCM/BG‐NCM extraction duration. (B) Cell counting analysis of U251 cells (B) and U87‐MG cells (C) in regular time point after the treatment of DMEM, DMEM + C‐NCM, DMEM + BG‐NCM, DMEM + C‐NCM + ADAM10i, and/or DMEM + BG‐NCM + ADAM10i (n = 6 independent cultures, *P < .05 vs the DMEM group). (D) Schematic illustration showing different colors represent different groups: black (DMEM group), orange (DMEM + C‐NCM), yellow (DMEM + BG‐NCM), purple (DMEM + C‐NCM + ADAM10i), and red (DMEM + BG‐NCM + ADAM10i). (E‐N) Cell count analysis of GBM001 (E), GBM002 (F), GBM003 (G), GBM004 (H), GBM005 (I), GBM006 (J), GBM007 (K), GBM008 (L), GBM009 (M), and GBM010 (N) in 0 and 24 h after treatment of DMEM, DMEM + C‐NCM, DMEM + BG‐NCM, DMEM + C‐NCM + ADAM10i, and/or DMEM + BG‐NCM + ADAM10i (n = 6 independent cultures, *P < .05 vs the DMEM group). The data are expressed as mean ± SE. Statistical analysis was implemented by student's *t* test and variance analysis

In the Figure 3C,F, we indicated that the levels of NLGN3 in the deep brain regions of normal human and mice brains are lower than those of the cortex. We know from previous studies that when patient‐derived GBMs are xenotransplanted into the frontal cortex of mice, GBM cells would grow.^20^ We can determine that the mouse's brain fluid environment is suitable for the growth of GBM cells. Neuroligin‐3 is highly expressed in neurons and oligodendrocyte precursor cells and is also secreted in both cells.^20^ We exfoliated the primary neurons of a fetal mouse's cortex tissue and basal ganglia tissue on the 18th day of pregnancy, respectively.^23^, ^24^ Then, we mixed the 9th day neuron culture medium (NCM) with DMEM to culture U251 cells and U87‐MG cells (Figure 7A). U251 cells and U87‐MG cells grew faster after mixing with NCM than with DMEM, and the cell density of the cortical neuron culture medium (C‐NCM) group was higher than that of the basal ganglion neuron culture medium (BG‐NCM) group at the same time after cell harvesting (Figure 7B,C). To further investigate whether NCM‐promoted cell growth is mediated by NLGN3, we used ADAM10 inhibitor (ADAM10i) GI254023X to inhibit the release of NLGN3.^20^ Neurons were treated with ADAM10i (200 nmol/L) after medium change on the seventh day (Figure 7A). We found that ADAM10i against C‐NCM‐ and BG‐NCM‐induced proliferation effect of GBM cells, consistent with previous research results (Figure 7B,C).^20^

We subcultured primary GBM cells from 10 different artificially numbered patients^25^, ^26^, ^27^: GBM001, GBM002, GBM003, GBM004, GBM005, GBM006, GBM007, GBM008, GBM009, and GBM010. Then, GBM001‐010 cells were treated in the same manner as shown in Figure 5A; different colors represent different groups (Figure 7D). In the Figure 7E‐N, the C‐NCM group and BG‐NCM group showed different degrees of promotion of GBM cell growth, and the proliferation effect was inhibited after treatment with ADAM10i. Therefore, higher levels of NLGN3 secreted by normal neurons promote a faster growth of GBM cells, and inhibition of NLGN3 release may be a target for inhibition of GBM growth.

## 4 DISCUSSION

Glioblastoma is the highest grade of brain gliomas.^28^ The median survival time after surgery is 14 months, followed by radiotherapy and/or chemotherapy. The main treatment of GBM is surgery, and short‐term recurrence is almost inevitable after surgery.^10^, ^29^ Preoperative assessment of GBM depends on MRI.^11^, ^30^ In our hospital, we conducted a retrospective study of 386 GBM cases from 2013 to 2016, of which 320 were followed up for more than 1 year and 66 cases failed to follow up. We found a meaningful outcome phenomenon. The recurrence of GBM mainly occurred in the basal ganglia, thalamus, and corpus callosum; however, the primary region mostly located on the cortex. To further investigate the recurrence of GBM, we followed up some of the patients who had undergone MRI after 1, 3, 6, 9, and 12 months of surgery. We have found that over time, recurrent GBMs grow into deeper brain regions and the cortex recurs. Previous articles on GBM have not found reports of this phenomenon.^28^, ^31^, ^32^, ^33^, ^34^

Neuroligin‐3 is a synaptic cell‐adhesion molecule that is present in both excitatory and inhibitory synapses and can interact with presynaptic neurexins and mediate transsynaptic signaling.^12^, ^35^ Recent studies have shown that NLGN3 is necessary for glioma growth, and GBM xenografts stopped growing in mouse brain after NLGN3 knockout.^20^ NLGN3 can also stimulate several pathways to promote gliomas proliferation.^36^ Therefore, we first tested the level of NLGN3 in normal human brains and found that NLGN3 levels in the basal ganglia, thalamus, and corpus callosum were higher than in the cortex, and similar patterns were observed in the mouse brain. Therefore, we further analyzed human GBM tissue from patients of surgery and found that NLGN3 levels were higher in the fundus region compared with that in the surface region. Furthermore, we tested the level of NLGN3 in normal tissues of GBM patients after tumor resection and found that NLGN3 levels are higher in deep brain regions, which is contrary to a normal human brain tissue. Thus, we speculate that primary gliomas mostly occur in the cortex and postoperative recurrence of GBM mainly in deep brain regions may have a strong correlation with NLGN3 levels, because NLGN3 is necessary for gliomas' growth. NLGN3 levels may be a potential factor in determining the recurrence of GBM.

To further verify our point of view, we used different concentrations of NLGN3 to treat U251 cells and U87‐MG cells,^37^ and analyzed the cell density at the time of onset after NLGN3 treatment. When the level of NLGN3 is higher, the functional coverage of the cell density of GBM is shown to be faster. To further validate our hypothesis, we used fetal mouse cortex and basal ganglia tissue to primary culture neurons, respectively.^23^ Then, we used the neuron culture medium of the 9th day (NCM) mixed with DMEM to culture U251 cells and U87‐MG cells. Results showed that the NCM group grew faster than the control group, and the C‐NCM group was the fastest group compared with the others. The neuronligin‐3 is cleaved from both neurons and oligodendrocyte precursor cells (OPCs) via ADAM10 sheddase, and the ADAM10 inhibitors can prevent the release of NLGN3, we used an ADAM10 inhibitor GI254023X to treat primary cultured neurons,^20^, ^38^, ^39^ the function of ADAM10 inhibitor is to prevent the release of NLGN3 into microenvironment.^20^ We did not find the proliferation effect of NCM in the NCM + ADAM10i group, the ADAM10i against NCM‐induced cell growth. Finally, we primary cultured the GBM cells, which were isolated from ten different patients of surgery, numbered GBM001‐010. And the results show similar patterns after processing with NCM and/or ADAM10i. Together, our data show that higher levels of NLGN3 promote faster growth of GBM cells, and inhibition of NLGN3 release may be a potential target to inhibit GBM growth.

## 5 CONCLUSIONS

Our findings demonstrate that the recurrence of GBM mainly occurs in the basal ganglia, thalamus, and corpus callosum, and the primary region of GBM mainly occurs in the cortex. We found that the level of NLGN3 is higher in the cortex than in the deep regions of a normal human brain. Interestingly, in GBM patients, NLGN3 levels were higher in deep brain regions than in the cortex. Increased concentrations of NLGN3 promote GBM cell growth in vitro. Inhibition of NLGN3 release prevents the proliferation effect of NLGN3. These results suggest that increased release of NLGN3 in deep brain regions may be involved in the recurrence of GBM after tumor resection.

## CONFLICT OF INTEREST

The authors declare that they have no conflict of interest.
