# Supplementary figures and images for: Glioblastoma recurrence correlates with NLGN3 levels
Source: Cancer Med. 2018 May 18;7(7):2848–59. doi: 10.1002/cam4.1538 (PMC6051187; doi:10.1002/cam4.1538)

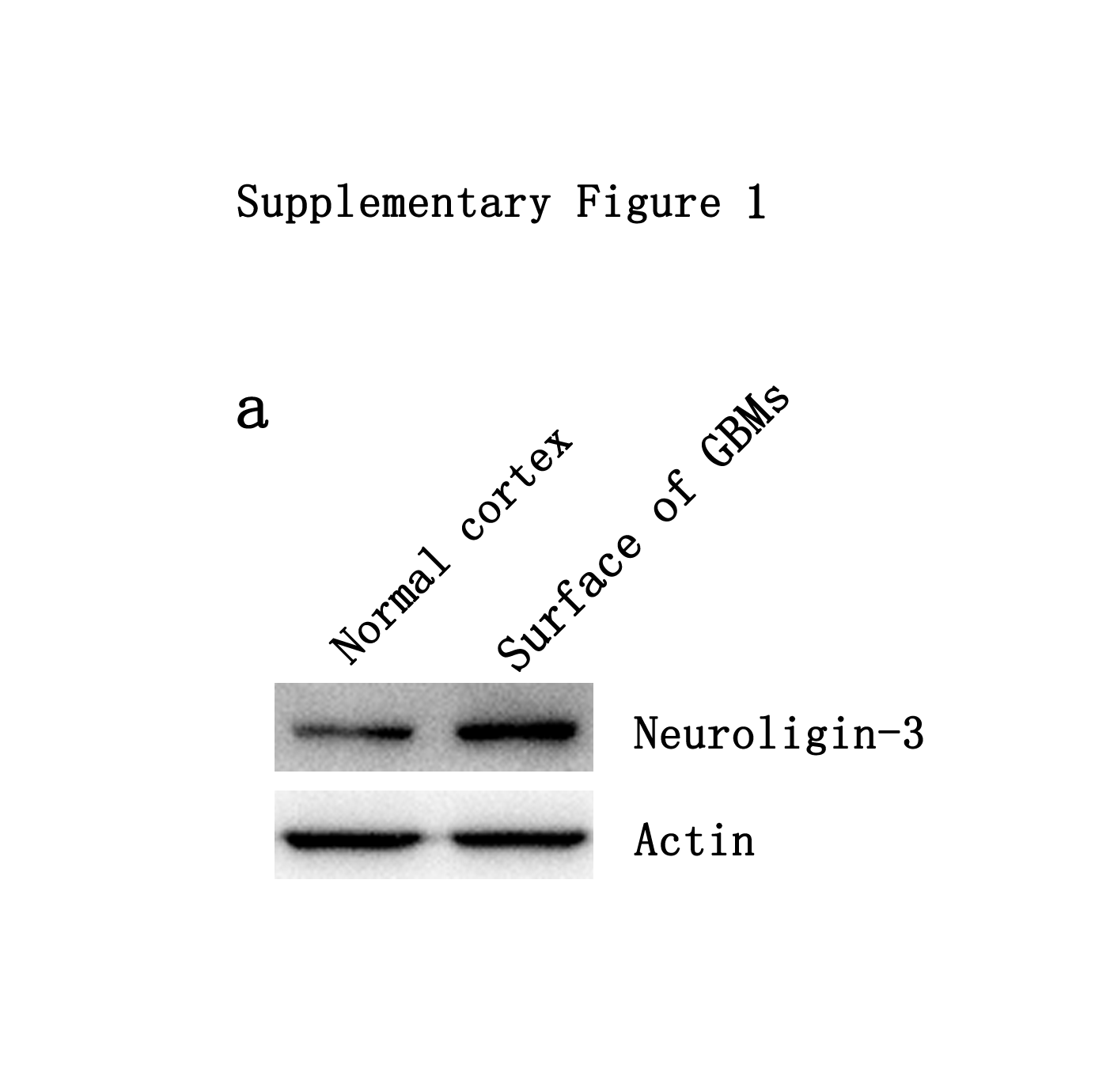

Supplement: Supplementary file 1 [file CAM4-7-2848-s001.tif]
